# PD-L1 expression, tumor-infiltrating lymphocytes, and mismatch repair proteins status in digestive neuroendocrine neoplasms: exploring their potential role as theragnostic and prognostic biomarkers

**DOI:** 10.1007/s00428-024-03825-5

**Published:** 2024-05-21

**Authors:** Eléonore Multone, Stefano La Rosa, Christine Sempoux, Silvia Uccella

**Affiliations:** 1 Institute of Pathology, Department of Laboratory Medicine and Pathology, University of Lausanne, Lausanne, Switzerland; 2 Unit of Pathology, Department of Medicine and Technological Innovation, University of Insubria, 21100 Varese, Italy; 3 Unit of Pathology, Department of Oncology, ASST Sette Laghi, Varese, Italy; 4 Department of Biomedical Sciences, Humanitas University, Milan, Italy; 5 Pathology Service, Istituti Di Ricovero E Cura a Carattere Scientifico (IRCCS) Humanitas Research Hospital, Milan, Italy

**Keywords:** PD-L1, Mismatch repair protein, Tumor immune infiltration, Neuroendocrine neoplasm, Digestive system, neuroendocrine tumors, neuroendocrine carcinomas.

## Abstract

Theragnostic biomarkers are still needed to select patients with digestive neuroendocrine neoplasms (NENs) for an optimal management. The PD-1/PD-L1 pathway plays a pivotal role in T cells activation and host immune response to cancer and PD-L1 expression in tumor and/or immune cells is used to identify patients who would benefit of treatment with immune checkpoint inhibitors. However, its role as a biomarker is still unclear in digestive NENs. We investigated PD-L1 expression in 68 well-characterized digestive NENs (32 NETs, 32 NECs and 4 MiNENs) and TPS and CPS scores were calculated. In addition, tumor infiltrating T-lymphocytes and mismatch repair protein expression (MMR) were evaluated. All results were correlated with clinicopathological features. PD-L1 expression was higher in NECs than in NETs: TPS > 1% and/or CPS > 1 were observed in 16% of NETs, 68.8% of NECs and 50% of MiNENs (p: 0.05). The mean TPS score in positive cases was 6.3% in NETs, 16.2% in NECs and 5% in MiNENs. The CPS score was 4.8 in NETs, 8.1 in NECs and 6 in MiNENs. MMR-deficient neoplasms were more frequently observed in NECs than in NETs (p: < 0.05) as well as intra-tumor immune infiltration (p: 0.00001). No correlation between PD-L1 expression and survival or other clinicopathological parameters was observed. Our results suggest that treatment with immune checkpoint inhibitors may have a potential role only in selected cases, mainly in NECs and MiNENs.

## Introduction

Gastroenteropancreatic (GEP) neuroendocrine neoplasms (NENs) encompass a heterogeneous group of malignant epithelial neoplastic proliferations including relatively indolent neuroendocrine tumors (NETs) and highly aggressive neuroendocrine carcinomas (NECs) [1]. NETs and NECs are two different diseases, with distinct morphological, molecular, and clinical features [2]. Based on their distinct biological features, they are treated differently in the context of clinically advanced disease. Generally speaking, platinum-based chemotherapy schedules are employed in patients with advanced NECs, whilst NETs can be managed with integrated approaches including surgery, somatostatin receptors targeted therapy, peptide receptor radionuclide therapy (PRRT), mTOR inhibitors (i.e., everolimus), antiangiogenic drugs (i.e., sunitinib), or chemotherapy depending on tumor type, grade, and stage [3–11]. However, despite the availability of multiple treatment options and guidelines from several scientific societies, there are still areas of controversy in advanced NENs treatment, for which there is limited guidance [12]. In this complex scenario, the role of immune checkpoint inhibitors (ICIs) therapy in NENs management has been preliminarily explored with conflicting and partially non-conclusive results and it still remains to be elucidated [13]. It is worth noting that the proper identification of patients to treat with ICIs reduces the cost/benefit balance, also in terms of adverse effects of these drugs, which may cause severe and potentially lethal conditions [14, 15]. Consequently, the evaluation of predictive biomarkers is needed to predict clinical benefit. High tumor mutational burden, the presence of mismatch repair protein (MMR) deficiency or of microsatellite instability, the estimate of tumor infiltrating lymphocytes (TILs), and the immunohistochemical analysis of PD-L1 expression on tumor cells and immune cells of the microenvironment are used as biomarkers for selecting patients receiving ICIs therapy [16].

Although some data have been published regarding lung NENs, studies exploring the clinical efficacy of PD-L1 inhibitors in extrapulmonary NENs have given conflicting results and the use of a combined or, mainly, single-agent does not seem to be particularly active for unselected patients [13]. In terms of biomarkers, it is worth noting that, although some studies suggest that PD-L1 is more frequently expressed by tumor cells in NECs than in NETs [17, 18], there are only few investigations exploring PD-L1 expression in association with the evaluation of TILs and microsatellite instability or mismatch repair (MMR) deficiency status, which are all associated with potential effectiveness of ICIs in re-activating anti-cancer immune response [19–22].

In this study we performed an integrated evaluation of PD-L1 and mismatch repair protein (MMR) immunohistochemical expression, together with TILs characterization in a series of well characterized digestive NENs with the aim of better defining the potential role of ICIs therapy in these diseases.

## Materials and methods

### Patients and samples

Sixty-eight consecutive GEP-NENs surgically resected between 2000 and 2010 were retrospectively selected from the files of the Institutes of Pathology of the University Hospitals of Lausanne (CHUV), Switzerland, and Varese (ASST Sette Laghi), Italy. For each patient the following clinico-pathological characteristics were collected from medical records: age, gender, tumor size, site, tumor stage and grade, and outcome. Only patients over 18 years of age and with at least 5 years of follow-up were included. Survival time was defined as the time between diagnosis and death or the date of the last follow-up control. All pathological diagnoses were reviewed and the 2019 WHO classification of digestive neuroendocrine neoplasms (5th edition) and the 8th UICC TNM classification were used for tumor grading and staging, respectively. Specimens resected in patients who underwent neo-adjuvant chemotherapy or radiotherapy were excluded. The dataset contained only de-identifiable data that were stored in an anonymous manner for protecting privacy and confidentiality.

### Morphological and immunohistochemical analyses

All tissues were fixed in buffered formalin and embedded in paraffin wax. 5 µm-thick sections were obtained from representative tumor blocks and were stained with hematoxylin–eosin (H&E) for the histopathological examination, which included the evaluation of morphological differentiation, mitotic count, presence of vascular, lymphatic and perineural invasion, and presence of necrosis. Immunohistochemistry (IHC) was performed on a Ventana Benchmark XT autostainer (Ventana Medical System, Tucson, AZ, USA) following the manufacturers’ guidelines and using the following antibodies: PD-L1 (monoclonal, clone SP263, Ventana), CD3 (monoclonal, 2GV6, Ventana), CD4 (monoclonal, clone SP35, Ventana), CD8 (monoclonal, clone C8/144B, Dako, Carpinteira, Denmark), hMLH1 (monoclonal, clone M1, Ventana), hMSH2 (monoclonal, clone G219-1129, Ventana), hMSH6 (monoclonal, clone 44, Ventana), hPMS2 (monoclonal, clone EPR3947, Ventana). Immunohistochemical stains for neuroendocrine markers (synaptophysin and chromogranin) and Ki67 were already available for all cases. The expression of immunohistochemical markers was evaluated as follows.

PD-L1 protein expression was evaluated by two pathologists (EM and SLR) and it was assessed according to the Combine Positive Score (CPS) and the Tumor Proportion Score (TPS) on four tumor fields at 200 × magnification that proportionally represented the various tumor staining areas and average of their scores was made. Tonsil tissue was used as normal control. The TPS is the ratio between PD-L1 positive tumor cells and the number of all viable tumor cells and is expressed as a percentage. The CPS was quantified by evaluating the number of PD-L1 positive cells (tumor cells, lymphocytes, macrophages) divided by the total number of viable tumor cells, multiplied by 100. Although the result of the calculation of CPS can exceed 100, the maximum score was defined as 100. Scores were given in whole numbers. In accordance with the criteria described in DAKO’s interpretation guide for CPS in head and neck squamous cell carcinoma, convincing partial or complete linear membrane staining (at any intensity and distinct from cytoplasmic staining) of viable invasive tumor cells was scored, whereas membrane and/or cytoplasmic PD-L1 staining was scored in the immune cells within the tumor nests and adjacent supporting stroma. Cases were considered PD-L1 positive when CPS score was > 1 and/or TPS score was > 1%.

Mismatch repair proteins expression was evaluated as positive (nuclear staining retained) or negative (global loss of nuclear staining with directly adjacent internal control). The loss of at least one of the MMR proteins was considered to define a neoplasm as MMR-deficient (MMRd).

The densities of CD3^+^, CD8^+^, and CD4^+^ lymphocytes were measured in four high power fields from each tumor by two experienced pathologists (EM and SLR) and the average density was calculated by counting the number of CD3, CD4 and CD8 positive lymphocytes in both the epithelial and stromal tumor components separately. Low infiltration of CD8^+^, CD3^+^ and CD4^+^ lymphocytes was defined as less than the median value.

The Ki67 proliferative index was expressed as the percentage of Ki67 immunoreactive cells on a total of 500–2000 neoplastic cells, manually counted on camera captured/printed images in the areas of higher nuclear labeling (“hot spots”). For the calculation of the Ki67 proliferative index, all the immunohistochemically labeled nuclei, regardless of the staining intensity or whether the nuclei show a speckled expression pattern or are diffusely stained, were considered for the scoring process.

### Statistical analysis

The Chi-square and Fisher exact tests were used to compare categorical variables and the Wilcoxon rank-sum test was used to compare continuous variables. Kaplan–Meier curves and log rank test were used to evaluate the prognostic impact of analyzed parameters. All statistical analysis was performed using SPSS. A p value < 0.05 was considered as statistically significant.

Data are presented as the mean ± SD. Statistical analyses and graphical representations were performed using SPSS 22.0 software (IBM Corp., Armonk, NY, United States) and GraphPad Prism 6 (San Diego, CA, United States) software, respectively. χ2 test and Fisher’s exact test were used to evaluate the correlation of PD-L1, CD3^+^, CD4^+^, CD8^+^, and MMR protein profile with clinico-pathologic parameters.

Survival curves were built using the Kaplan–Meier method and differences were analyzed by the log-rank test. Identification of factors that had a significant influence on survival was performed by univariate and multivariate Cox regression analyses. Comparison between two groups was performed by the Student’s t-test or Mann–Whitney U test. P-values (two-sided) < 0.05 were considered statistically significant.

## RESULTS

### Clinico-pathologic features

Of the 68 GEP-NENs included in the study, 32 were NECs, 4 MiNENs, and 32 NETs, including 22 NETs G1, 9 NETs G2, and 1 NET G3. Forty-three patients (63.2%) were males (21 with NET, 18 with NEC, 4 with MiNENs) and 25 (36.8%) were females (11 with NET, 14 with NEC). The mean age at diagnosis was 62.6 years (median = 65.5 years, age range 19–83 years). Most of the neoplasms (65/68, 95.6%) were nonfunctioning; only 3 NETs were associated with Cushing, insulinoma, and Zollinger Ellison syndrome, respectively.

Regarding the site of origin, 12 neoplasms were located in the pancreas (11 NETs and 1 NEC), 15 in the stomach (3 NETs and 12 NECs), 23 in the large bowel (19 NECs and 4 MiNENs), 15 in the small intestines (all were NETs) and 3 in the appendix (all were NETs). 47/66 (71.2%) tumors were metastatic at diagnosis, 45 with nodal (17 NETs, 24 NECs, 4 MiNENs), 12 with distant (7 NETs and 5 NECs), and 10 with both nodal and distant metastases (6 NETs and 4 NECs). For 3 NECs the nodal status was not available but one of them was positive for distant metastases. The T-classification at diagnosis was T1 for 10 neoplasms (9 NETs, 1 NEC), T2 for 14 (5 NETs, 9 NECs), T3 for 32 (10 NETs, 18 NECs, 4 MiNENs), and T4 for 11 (8 NETs, 3 NECs). For 1 NEC, T information was not available. Tumor stage was I for 9 neoplasms (9 NETs), II for 12 neoplasms (5 NETs, 7 NECs), III for 34 neoplasms (11 NETs, 19 NECs, 4 MiNENs), and IV for 12 neoplasms (7 NETs, 5 NECs). For 1 NEC, tumor stage was not available.

Among the 32 NECs, 2 (6.25%) were classified as small cell neuroendocrine carcinoma (SCNEC) and 30 (93.75%) as large cell neuroendocrine carcinoma (LCNEC). In six of them a non-neuroendocrine component ranging between 5 to 20% was observed. These cases were not diagnosed as MiNENs following the strict criteria of the WHO classification.

Four cases were diagnosed as MiNENs because both the neuroendocrine and non-neuroendocrine components reached > 30% of the tumor burden. In one out four cases the neuroendocrine component was represented by SCNEC while in the other 3 by a LCNEC. The neuroendocrine components of MiNENs showed the same morphological features and immunohistochemical profile observed in small cell and large cell NECs when presenting as pure NECs. The non-neuroendocrine component was represented by an adenocarcinoma in all cases.

The median Ki67 index was 2% (range: 1–25%) for NETs, 70% (range: 23–90%) for NECs and 55% (range: 50–70%) in the neuroendocrine components of MiNENs.

### MMR analysis

**Fig. 1:**
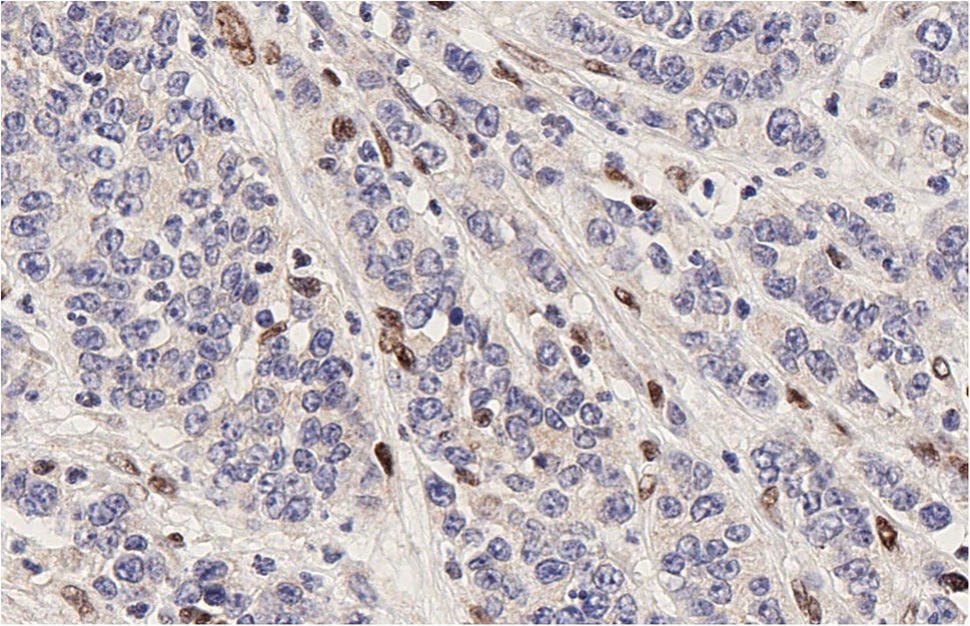
MLH1 immunoreactivity is lacked in tumor cells of this colonic neuroendocrine carcinoma. Interstitial lymphocytes are positive representing normal internal control

MMR immunohistochemical analysis allowed us to identify 7/68 (10.3%) MMR-deficient (MMRd) tumors: five NECs and 2 NETs (p: < 0.05). All of the 7 cases were lacking hPMS2 expression and 5 of them also showed a concomitant loss of hMLH1 (4 NECs and 1 NET) (Fig. 1). All MiNENs were MMR-proficient (MMRp). No correlation was found between MMRd and other clinico-pathological parameters.

### PD-L1 expression in NETs and NECs

**Fig. 2:**
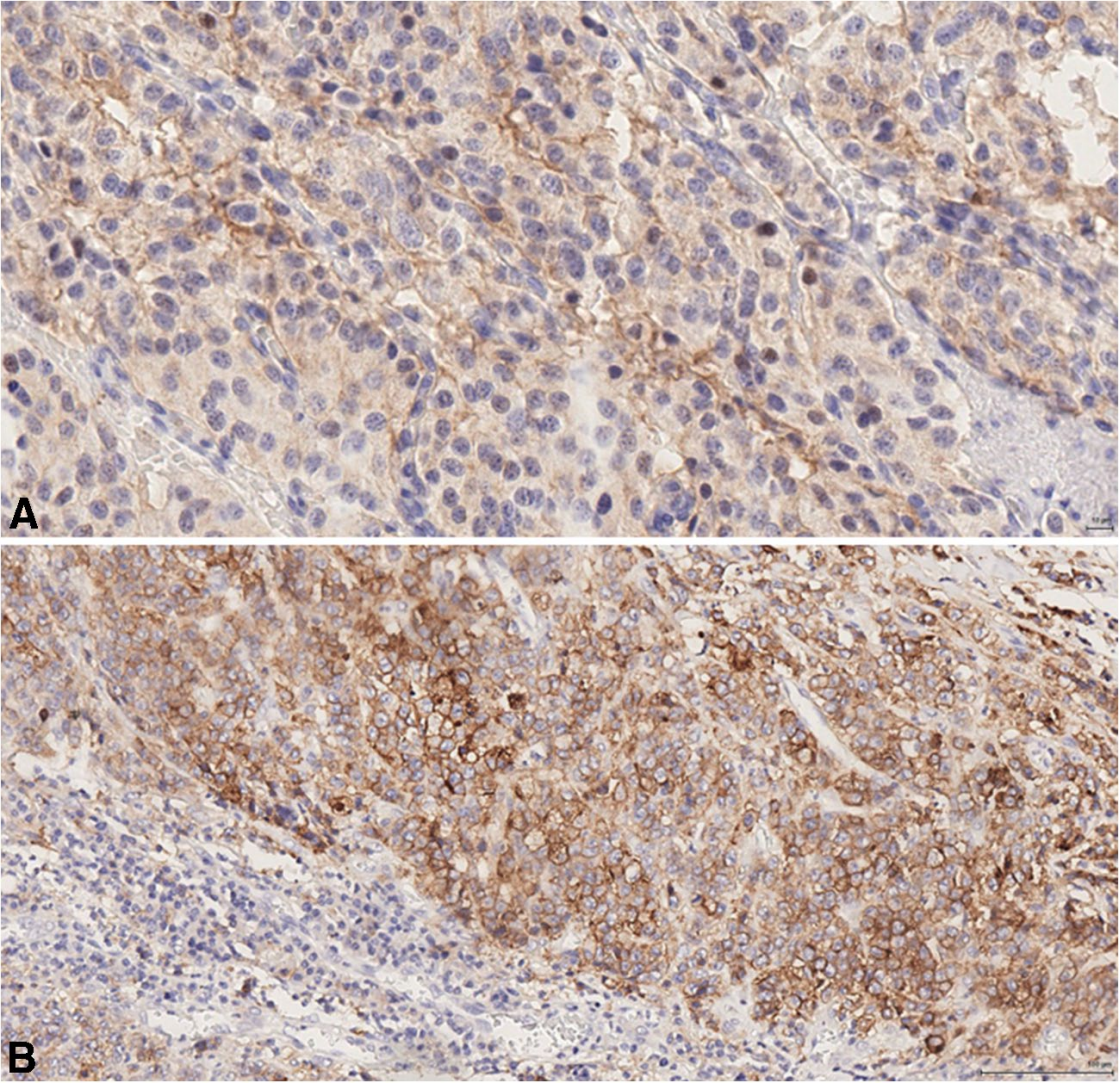
PD-L1 expression was observed more frequently in NECs than in NETs. In positive cases both the mean TPS and CPS scores were lower in NETs (**A**) than in NECs (**B**)

PD-L1 expression (determined as scores TPS > 1% and/or CPS > 1) was observed in 5/32 (16%) NETs, 22/32 (68.8%) NECs and 2/4 MiNENs (50%) (p: 0.05). The mean TPS score in positive cases was 6.3% in NETs (range: 2–14, median = 3), 16.2% in NECs (range: 3–52, median = 5) and 5% in MiNENs (only one case) (Table 1 and Fig. 2). The mean CPS score was 4.8 in NETs (range: 2–14, median = 3), 8.1 in NECs (range: 2–54, median = 4) and 6 in MiNENs (range 2–10, median = 6). The five PD-L1 positive NETs showed a CPS > 1 and three of them also showed a TPS > 1%. The 21 positive NECs had a CPS > 1 and six of them also presented a TPS > 1%. The 2 positive MiNENs had a CPS > 1 and only one also presented a TPS > 1%. PD-L1 expression alone did not correlate with survival or other clinico-pathological parameters, including Ki67 proliferative index and MMR protein deficiency (Table 1).

**Table 1:** Clinico-pathologic features of PD-L1 positive and negative neoplasms (n total = 68)

Variables	NET PD-L1 neg (N = 27)	NET PD-L1 pos (N = 5)	NEC PD-L1 neg (N = 11)	NEC PD-L1 pos (N = 21)	MiNEN PD-L1 neg (N = 2)	MiNEN PD-L1 pos (N = 2)
Gender						
Male	18	3	8	10	2	2
Female	9	2	3	11	0	0
Age at diagnosis						
Median (range)	64 (19–86)	61 (22–65)	67 (52–82)	68 (34–82)	65.5 (56–75)	76.5 (70–83)
WHO classification						
NET						
G1	19	3				
G2	7	2				
G3	1	0				
NEC						
Large cell			10	19	2	1
Small cell			1	2	0	1
Primary tumor site						
Pancreas	8	3	0	1	0	0
Stomach	3	0	2	10	0	0
Large bowel	0	0	9	10	2	2
Small intestine	13	2	0	0	0	0
Appendix	3	0	0	0	0	0
TNM classification						
T1	8	1	1	0	0	0
T2	5	0	1	8	0	0
T3	8	2	6	12	2	2
T4	6	2	3	0	0	0
n.a	0	0	0	1	0	0
N0	14	1	0	5	0	0
N1	13	4	10	14	2	2
n.a	0	0	1	2	0	0
M0	21	4	9	18	2	2
M1	6	1	2	3	0	0
Stage						
I	8	1	0	0	0	0
II	5	0	0	7	0	0
III	8	3	9	10	2	2
IV	6	1	2	3	0	0
n.a	0	0	0	1	0	0
Survival (months)						
Mean	60	60	26	50	1.5	4.5
Ki-67 (%)						
Mean	3.9	5.6	70	61	55	55
Median (range)	2 (1–25)	2 (2–12)	70 (40–90)	70 (23–90)	55 (50–60)	55 (50–60)
MMR-Status						
MMRp	25	5	10	17	2	2
MMRd	2	0	1	4	0	0
CPS						
Mean		4.8		8.1		6
Median (range)		3 (2–14)		4 (2–54)		6 (2–10)
TPS		N = 3		N = 5		N = 1
Mean		6.3		16.2		5
Median (range)		3 (2–14)		5 (3–52)		5
Functioning	2	1	0	0	0	0

NET: neuroendocrine tumor; NEC: neuroendocrine carcinoma; n.a.: unknown

### Tumor-infiltrating lymphocytes (TILs)

**Fig. 3:**
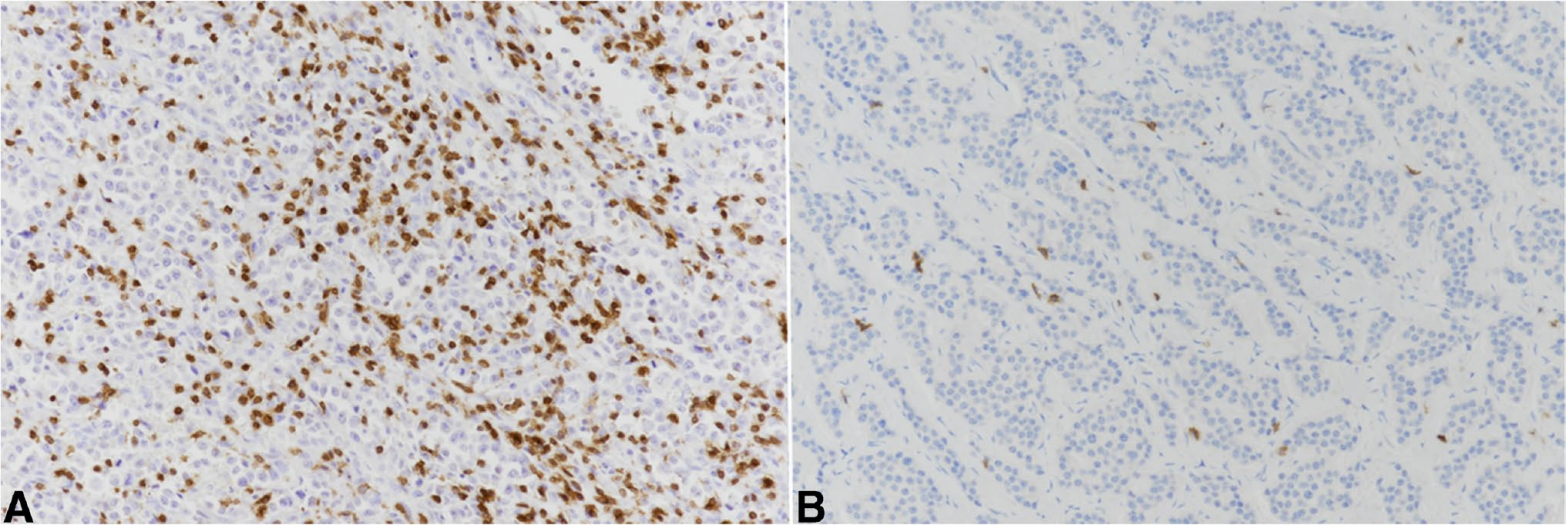
Tumor-infiltrating (intra-stromal and intra-epithelial) CD3 + T-lymphocytes were significantly more abundant in NEC (**A**) than in NET (**B**)

Tumor-infiltrating (intra-stromal and intra-epithelial) CD3^+^ T-lymphocytes were significantly more abundant in NECs than in NETs (p = 0.00001) (Fig. 3), but it did not show any difference between MMRd and MMRp cases or with other evaluated parameters. Non-significant differences were observed in CD4^+^/CD8^+^ ratio between NETs and NECs.

### Survival analysis

The mean overall survival time was 82 months for NETs (range: 28–140), 43 months for NECs (range: 1–257) and 3 months for MiNENs (range: 1–5). 29/67 (43.3%) patients died of disease (2/32 NETs, 23/31 NECs and 4/4 MiNENs), 9/67 (13.4%) died of other causes (5/32 NETs and 4/31 NECs), 6/67 (9%) were still alive with disease 5 years after diagnosis (only NETs), and 23/67 (34.3%) were alive without disease 5 years after the diagnosis (19/32 NETs, 4/31 NECs). One NEC was lost to follow-up.

Univariate survival analysis performed in the NECs cohort showed that MMR deficiency and a moderate-to-intense lymphoid infiltrate were significantly related to a longer overall survival. Specifically, patients with MMR-proficient neoplasms had a median OS of 7.7 months, versus 73.4 months of patients with MMR-deficient NECs (p = 0.04; 95% confidence interval: -0.2158 to -0.3865); patients with moderate-intense lymphoid infiltrate had a median OS of 6.3 months, versus 23,4 months of patients with mild lymphoid infiltrate (p = 0.008: 95% confidence interval: -2324 to 0.5803). In turn, PD-L1 expression (CPS > 1 or TPS > 1%) was not related to a different outcome neither for NETs nor for NECs and the composition of T-cell infiltrate in terms of prevailing CD8 + or CD4 + cells did not prove to be a prognostic factor.

## Discussion

ICIs-based therapeutic approach is now accepted for several different cancers including melanoma, head and neck squamous cell carcinoma, lung cancers including small cell carcinoma, renal cell carcinoma, urothelial carcinomas, and others [14]. The clinical efficacy of ICIs therapy reflects the immunomodulatory effect mediated by the binding with their specific antigens involved in the regulation of immune response, consequently increasing host response to tumor cells [16]. It clearly appears that ICIs performance depends on the correct selection of patients who can benefit from the therapy, excluding those potentially not responding for whom the cost/benefit of ICIs therapy, which is not free of adverse symptoms (including, but not limited to, hypophysitis, thyroiditis, and colitis), is not favorable [15].

The usefulness of ICIs for the therapy of digestive NENs remains to be established, as published results are conflicting [13, 23]. The objective response rates in patients treated with pembrolizumab, spartalizumab, avelumab or combined nivolumab/ipilimumab, durvalumab/tremelimumab, or atezolizumab/bevacizumab are variable from 0% to 3.7% in unselected patients. It has been observed that only 10–30% of digestive NENs express PD-L1 [17–19, 24] and the need for a proper selection of patients undergoing ICIs treatment may be advocated on these bases. Indeed, besides PD-L1 expression, also MMRd and intense intratumor lymphoid infiltrate have been used as predictors of good response to ICIs [25–32].

In our series, we have found PD-L1 expression in 28 out of 68 NENs, representing 41.2% of cases. Considering studies published in the literature, PD-L1 expression has been reported in 34.2% GEP-NENs, with a range from 6.1% to 75% (Table 2). Although the specific comparison of our findings with those of other studies is not easy due to the different antibodies used, different methods of evaluation employed, and different NEN classifications considered, the percentage of positive cases we observed is generally comparable with the literature data. For the first time in GEP-NENs, we evaluated and compared both TPS and CPS scores, which represent two different systems for reporting PD-L1 expression in tumors. TPS score refers to PD-L1 expression only in tumor cells and is mainly used in the lung to select patients eligible for ICIs therapy [33–35]. The CPS score includes the evaluation of PD-L1 expression in both neoplastic and inflammatory cells and is generally used in cancers arising in the head and neck region or in urothelial mucosa [36–38]. A comparison between TPS and CPS scores has never previously been reported in digestive NENs and the use of ICI has only been used in advanced with limited response and poor connection with PD-L1 expression in immunohistochemistry [31, 39–44]. In our series, we have found more tumors with CPS > 1 than with TPS > 1% and it is conceivable that CPS may be proposed as the standard score for determining PD-L1 positivity in NENs for treatment with ICIs, although specific clinical trials are needed to determine which of the two scoring systems is truly more effective. As expected, the number of PD-L1 positive cases (considering both TPS and CPS) was higher among NECs than NETs (p = 0.003). Additionally, we observed a direct correlation between PD-L1 positive cases and proliferation grade of in NETs, in line with previously published studies [45–48].

**Table 2:** Summary of literature data on PD-L1 expression in digestive neuroendocrine neoplasms

Reference	Number of cases studied	Number PD-L1 + NETs (%)	Number PD-L1 + NECs (%)
Rosery et al. 2021 [49]	**37** *GEP NET G3* + *NEC*		14 (38%)
Roberts et al. 2017 [50]	**37** *GEP NEC* • **12** *SCNEC* • **25** *LCNEC*		12 (32%) • 6 (50%) • 6 (24%)
Cives et al. 2019 [51]	**102** *Si-NET* • **94** *G1* • **8** *G2*	40 (39%) • 37 (39.4%) • 3 (37.5%)	
Yang et al. 2019 [52]	**43** *G-NEC*		21 (48.8%)
Xing et al. 2020 [27]	**31** *GEP NET G3* + *NEC*		9 (29%)
Rösner et al. 2022 [53]	**175** *GEP NET* • **79** *G1* • **67** *G2* • **23** *G3*	n.r. (73%) • n.r. (58.2%) • n.r. (83.1%) • n.r. (86.4%)	
Bösch et al. 2019 [25]	**231** *GEP NET* • **213** *G1/G2* • **18** *G3*	20 (8.7%) • 17 (8%) • 3 (18%)	
Wang et al. 2019 [54]	**120** *GEP NET* • **53** *G1/G2* • **67** *G3*	63 (52.5%)	
Oktay et al. 2018 [55]	**59** *GEP NET* • **27** *G1* • **20** *G2* • **12** *G3*	7 (11.9%) • 1 (3.70%) • 2 (10%) • 4 (33.3%)	
Lamaraca et al. 2018 [19]	**70** *Si-NET* • **47** *G1* • **23** *G2*	21 (30%)	
Ali et al. 2020 [56]	**136** *GEP NET G3*	14 (10.%)	
Hasewaga et al. 2021 [57]	**20** *GEP NEN* • **6 ***NET G1* • **8** *NET G2* • **6** *NEC (4 SCNEC, 2 LCNEC)*	6 (100%) 6 (75%)	3 (50%)
Yamashita et al. 2020 [58]	**25*** G-NEC (10 pure NEC****,**** 15 mixed GAC-NEC, 10 SCNEC, 15 LCNEC)*		18 (72%)
Kim et al*.* 2016 [18]	**32** *GEP NET* • **15** *G2* • **17** *G3*	7 (21.9%) • 0 • 7 (21,9%)	
Sampedro-Núñez et al. 2018 [45]	**116** *GEP NET*	7 (6.1%)	
Cavalcanti et al. 2017 [17]	**57** *GEP NET* • **39** *G1* • **9** *G2* • **9** *G3*	16 (28%) • 0 • 7 (78%) • 9 (100%)	

Si-NET: small-intestinal neuroendocrine tumor; SCNEC: small cell neuroendocrine carcinoma; LCNEC: large cell neuroendocrine carcinoma; G-NEC: gastric neuroendocrine carcinoma; n.r.: not reported; GAC: gastric adenocarcinoma

For the analysis of tumor infiltrating lymphocytes (TILs), we counted both intra-epithelial and intra-stromal CD3^+^ T lymphocytes. In addition, the number of CD4^+^ and CD8^+^ T-cells was evaluated. The CD3^+^ TILs counts were significantly higher in NECs than in NETs (p = 0.00001 for intra-stromal TILs and p = 0.001279 for intra-epithelial TILs). CD4^+^/CD8^+^ ratio was not significantly different between NECs and NETs; moreover, we did not find a definite predominance of CD4^+^ or CD8^+^ cells in TILs. The more abundant lymphocytic infiltration observed in NECs than in NETs is in line with previous findings that also demonstrated a direct correlation with PD-L1 expression, not observed in our study [45, 54, 59]. Because of the immunological implications, high TIL count has been considered a prognostic marker to select patients responding to checkpoint inhibitors therapy, although a consensus has not been still reached, especially for digestive NENs [19, 56]. However, the results of our study demonstrating that NECs generally show a more abundant lymphocytic infiltration than NETs suggest that immunological therapies may be considered only for this aggressive NEN subtype.

Intratumor inflammatory infiltration has more frequently been observed in MMRd and/or MSI NENs [60, 61] and for this reason we evaluated the expression of mismatch repair proteins in our NENs using immunohistochemistry. Seven out of 68 cases showed MMRd and were predominantly represented by NECs (p < 0.05). In our series, we did not find any correlation between MMRd and other clinico-pathological parameters when considering both NETs and NECs. The observation that MMRd is more frequently observed in NECs, which also show a higher intratumor lymphocytic infiltration, and that is associated with better prognosis is in line with previous data [21] supports the hypothesis that immunological therapies should be employed in a subset of NECs.

Finally, we correlated PD-L1 expression with other clinico-pathological parameters (age, gender, histological subtype, etc.…) and we did not find any relevant correlation. Survival was not related to PD-L1 expression, which was one of our starting hypotheses. A correlation between PD-L1 expression and survival has been reported in only one study [18], while it was not found in others [48, 62, 63]. Although PD-L1 expression alone cannot be considered as a prognostic marker, its prognostic role may change in future if it can be used to select patients potentially responding to ICIs therapy. Consequently, its predictive role may influence the prognosis of patients with NECs. This may open a new therapeutic strategy, considering that digestive NECs are currently treated with platinum-based chemotherapy, which has not significantly increased patient’s survival in the last years [64].

We are well aware that, like all retrospective studies, our investigation shows potential limitations. Specifically, staging system procedures (i.e., radiology and/or nuclear medicine investigations, and laboratory tests) and therapeutic protocols were not the same for all patients since the diagnoses were performed in a range of 10 years, a long period in which new clinical and therapeutic approaches have been developed. In addition, not all patients were treated in the same hospital or by the same team. Nevertheless, to reduce biases, all tumors were reclassified according to the 2019 WHO classification of digestive neuroendocrine neoplasms.

In conclusion, this study, which should be considered as a preliminary investigation, has demonstrated that PD-L1 expression is not correlated with clinical and pathological data. Thus, it cannot be considered a significant biomarker on its own. However, the heterogeneous expression of PD-L1 in NENs, with a clear predominance in NECs and in high grade NETs, may suggest selecting specific patients for whom targeted therapy with checkpoint inhibitors may have a beneficial effect. In our hands, the CPS score proved to be more sensitive than TPS in identifying PD-L1 positive cases and a value of CPS > 1 may be suggested, at this stage, as potentially predictive of a good response to ICIs. Furthermore, the T-cell infiltrate and MMRd, more frequently observed in NECs than in NETs, further support the higher efficacy of immunological therapy in NECs.

This study, although with the limitations above discussed, compares for the first time PD-L1 expression (using both CPS and TPS scores), tumor infiltrating lymphocytes, and MMR status in NENs of the digestive tract, including NETs and NECs. Our results provide a conceptual rationale for restricting the employment of immune therapy to NECs, in which this kind of therapeutical approach is likely to be more effective.

## Declarations

This study was performed according to the clinical standards of the 1975 and 1983 Declaration of Helsinki and was approved by the local ethical committee (Comité d’éthique sur la recherche humaine du canton de Vaud, ref ID 2019–02259).
